# Patient-facing genetic and genomic mobile apps in the UK: a systematic review of content, functionality, and quality

**DOI:** 10.1007/s12687-022-00579-y

**Published:** 2022-02-19

**Authors:** Norina Gasteiger, Amy Vercell, Alan Davies, Dawn Dowding, Naz Khan, Angela Davies

**Affiliations:** 1 Division of Nursing, Midwifery and Social Work, School of Health Sciences, University of Manchester, Manchester, UK; 2 Division of Informatics, Imaging & Data Sciences, School of Health Sciences, The University of Manchester, Manchester, UK; 3 The Christie NHS Foundation Trust, Wilmslow Road, Manchester, M20 4BX UK; 4 Manchester Centre for Genomic Medicine, St. Mary’s Hospital, Manchester Academic Health Science Centre, Manchester University NHS Foundation Trust, Manchester, M13 9WL UK; 5 Public Health Department, Blackburn With Darwen Borough Council, Blackburn, BB2 1DH UK

**Keywords:** Genetics, Genes, Genomics, Smartphone app, App review

## Abstract

Close relative (consanguineous) marriage is widely practised globally, and it increases the risk of genetic disorders. Mobile apps may increase awareness and education regarding the associated risks in a sensitive, engaging, and accessible manner. This systematic review of patient-facing genetic/genomic mobile apps explores content, function, and quality. We searched the NHS Apps Library and the UK Google Play and Apple App stores for patient-facing genomic/genetic smartphone apps. Descriptive information and information on content was extracted and summarized. Readability was examined using the Flesch–Kincaid metrics. Two raters assessed each app, using the Mobile App Rating Scale (MARS) and the IMS Institute for Healthcare Informatics functionality score. A total of 754 apps were identified, of which 22 met the eligibility criteria. All apps intended to inform/educate users, while 32% analyzed genetic data, and 18% helped to diagnose genetic conditions. Most (68%) were clearly about genetics, but only 14% were affiliated with a medical/health body or charity, and only 36% had a privacy strategy. Mean reading scores were 35 (of 100), with the average reading age being equivalent to US grade 12 (UK year 13). On average, apps had 3.3 of the 11 IMS functionality criteria. The mean MARS quality score was 3.2 ± 0.7. Half met the minimum acceptability score (3 of 5). None had been formally evaluated. It was evident that there are few high-quality genomic/genetic patient-facing apps available in the UK. This demonstrates a need for an accessible, culturally sensitive, evidence-based app to improve genetic literacy within patient populations and specific communities.

**Supplementary Information:**

The online version contains supplementary material available at 10.1007/s12687-022-00579-y.

## Introduction

In 2020, analysis of the Orphanet rare disease registry found there to be 6172 unique rare diseases, and 71.9% of them were genetic; the global prevalence of rare disorders equates to between 263 and 446 million people affected at any one time (Nguengang Wakap et al. 2020). A genetic variant is defined as a heritable change to the DNA sequence, which can be benign or pathogenic and involves a single gene or multiple genes (Richards et al. 2015). The impact of these genetic variants can vary from having a positive impact by keeping populations healthy, to no or minimal implications upon a person’s health or development, to significant morbidity and mortality (Eichler 2019). In the UK, 30,000 children receive a diagnosis of a genetic condition each year, and more than half a million children and adults are living with a genetic disorder (Gene People 2020; Genetic Alliance 2021).

Many West and South Asian countries have a very high prevalence of consanguineous marriage, especially close-cousin marriage (Becker et al. 2015; Hussain 2002). Roughly 20% of the world’s population (as of 2002) live in communities with a preference for consanguineous marriage (Modell and Darr 2002), with 10.7% (as of 2015) of the world’s population being consanguineous (Becker et al. 2015). Consanguineous marriage is also common in the UK among the Pakistani community, which as of 2016 had numbers over 1.1 million (Bittles and Small 2016; Khan et al. 2016), with roughly 59.3% of women in the UK Pakistani community married to first or second cousins (Bhopal et al. 2014).

Due to the rates of consanguineous marriage among the Pakistani community, the risks of recessive genetic disorders are higher than in non-consanguineous populations (Khan et al. 2010; Posch et al. 2012; Shaw and Hurst 2008). An average non-consanguineous couple will have a baseline risk of 2–3% for having a child with a genetic disorder; for a consanguineous couple, an additional 2–4% risk should be added (Teeuw et al. 2014). The closer the relationship, the greater proportion of genes shared by the couple and the greater the risk that the offspring will be homozygous for the shared gene (Harper 2004). Such recessive disorders contribute to increased rates of infant mortality and morbidity in England (Salway et al. 2016).

The Born in Bradford (UK) cohort study prospectively recruited 12,453 women from white British and Pakistani (and other ethnicity groups) to complete a questionnaire relating in part to consanguinity within their relationship and their social, economic, and health factors (Bhopal et al. 2014). Unsurprisingly, consanguinity rates were much higher in the Pakistani group compared to the white British group (37.5% vs. 0.0% for first-cousin marriage). Mothers within a consanguineous relationship were more likely to be less educated than those in non-consanguineous relationships (19.2% vs. 35.2% A-level attainment, PR = 0.7). Fathers in consanguineous relationships were also more likely to be less educated and more likely to be in routine manual jobs. Consanguineous couples overall were more likely to be living in the lower index of multiple deprivation areas compared to their non-consanguineous counterparts.

A lack of access to culturally sensitive and accessible genetic information means that there is a significant unmet need in terms of educating Pakistani communities in the UK regarding genetics and providing signposting and access to clinical genetics services. Consanguineous couples should receive appropriate information regarding their increased reproductive risk and possibilities for genetic counselling (Teeuw et al. 2014).

The authors of this study are working with the South Asian Pakistani community in Blackburn with Darwen in the UK to co-create an educational intervention to improve genetic literacy within the community. The creation of a mobile app that addresses poor genetic literacy may promote empowerment and the ability for individuals to make more informed decisions regarding marriage and childbearing, which has the potential to reduce the incidence of genetic disorders within this population. Initial findings from this study (including semi-structured interviews with 7 community members) indicated that a mobile app could be used within this community and that it is likely that younger people would access this information ahead of planning for their families or even premarriage (unpublished findings). Interview respondents indicated a likelihood that they would use this app either alone or with older family members to help to educate them and make informed choices. The authors’ initial findings also highlighted other specific user requirements including privacy, lack of obvious connection to genetics in the app branding, and the requirement for high quality and trusted branding (e.g. NHS logo).

Smartphones are an increasingly used platform, with the potential to change health-related behaviours. However, whilst the number of mobile phone health apps are vast, engagement varies for a myriad of reasons. A population-based survey (*n* = 4144) among German people who were aged 35 years or older found that 61% of participants used a smartphone, with users being younger, more likely to be educated to a university degree, more likely to work full-time, and be more engaged with health-related quality of life and health literacy (Ernsting et al. 2017). A cross-sectional survey of 1604 mobile phone users in the USA found that 58% of users had downloaded a health-related mobile app, but 45.7% of them failed to engage in continued use due to high data entry burden, loss of interest, and hidden costs (Krebs and Duncan 2015). These socioeconomic, age, and literacy-related factors highlight disparities in the use of mobile technology. Similarly, the quality and credibility of any health-related app is of utmost importance, with peer-reviewed, evidence-based products being more likely to gain users whilst maintaining user safety. Involving patient groups and communities in the development and testing of mobile medical apps is also beneficial (Ceasar et al. 2019; Derbyshire and Dancey 2013).

A previously conducted systemic app review on 88 genetic apps available in the USA found that 55.7% (*n* = 49) were created by commercial companies, and only 13.6% (*n* = 12) were created by a reliable or credible agency (Talwar et al. 2019). These apps were also predominantly focused on health professional students rather than patients (86.4%, *n* = 74), and over one-third of the apps did not have a customer rating. Whilst the apps had the potential to promote healthy behaviours based on genetic information, the accuracy and evidence of their recommendations are unclear. The authors also expressed concerns regarding the quality of the apps overall. The present systematic app review will focus on identifying specifically patient-facing smartphone apps related to genetic or genomic conditions available in the UK and explore their purpose, functions, and quality, thereby feeding into the proposed design of the aforementioned app. This review is part of a larger research study, which underpins the design of the mobile app, also including interviews with community members to define user requirements and a systematic review of the literature. It is hoped that this three-pronged approach will help us to define the required content and also the best way to present this within the app.

## Methods

A systematic app review and content synthesis was conducted on genomic/genetic apps available in the UK. Where applicable, the review aligned with the Preferred Reporting Items for Systematic Reviews and Meta-Analysis (PRISMA) guidelines (Moher et al. 2009).

### Search strategy and inclusion criteria

Apps were identified from the NHS App Library and the UK Google Play and Apple App stores from the 28th of June until the 1st of July 2021. Keywords for the search included: genetic, genetic condition/s, genetic disease/s, DNA, genome, genomic, genomic condition/s, and genomic disease/s.

Patient-facing apps that focussed on genetic conditions (for any purpose) and were aimed at adults (18 years or older) were included. Apps were included regardless of whether they were aimed at groups or individuals. Apps covering other health conditions (e.g. infectious diseases) not in English and those aimed at children, healthcare providers, organisations, and students were excluded. Apps were also excluded if they required external devices (e.g. wearables, virtual reality headsets, or smartwatches) or if they relied on DNA kits/samples or genetic counselling to login.

### App selection

We selected relevant apps through a two-step process. Two authors (AV and NG) first screened the app markets by reading relevant app names and descriptions. Duplicates were removed between the markets and the individual searches. Second, three authors (NG, AV, and AD) downloaded the apps and screened them for eligibility. Two researchers reviewed each app, whereby two (NG and AV) reviewed those in the Apple App store using the iPhone SE (iOS 13.6.1) and iPhone 12 Pro (iOS 14.7.1) devices. Two researchers (NG and AD) reviewed those available in the Google Play store using the Samsung Galaxy A41 (Android 10 with One UI 2.0) and Samsung Galaxy S7 (Android 8.0.0).

### Data extraction

Informed by previous app reviews (Ali et al. 2020; Gasteiger et al. 2021), a coding sheet was created on Microsoft Excel, into which three raters (AV, NG and AD) extracted data from the apps (see Table 1). Descriptive information included the app’s name, developer, version number, the app market/s in which it was available, cost to download, whether it was affiliated with a professional health/medical body or charity, average user rating, and the number of user ratings. We assessed descriptive technical content by determining whether apps contained a privacy strategy, mentioned third-party authorisations, and whether they asked to work in the background, worked offline, or were asked to enable push notifications.

**Table 1:** Description of the data extraction items

Items	Description
Descriptive information	
App name	Name of the mobile app
Version number	Version of the app reviewed
Developer	Name of developer
Market/s available	Google Play; Apple App; NHS Apps Library
Cost	Free to download, cost to download (in GBP); in-app purchases
Affiliated with a professional medical/health body or charity	Yes; no
Average user rating	Not rated; average number of public ratings (maximum 5 points)
Number of user ratings	Total number of user ratings
Privacy strategy	Privacy policy, login, password, two-factor authentication
Third-party authorizations (e.g. data sharing)	Yes; no
Works offline	Yes; no
Works in the background	Yes; no
Asks to enable push notifications	Yes; no
Content	
Purpose	Diagnose, record data/track, educate/inform, instruct, remind, analyse (i.e., DNA sample/test data)
Description	Summary of the app’s content
Obviously about genetics (considering name and icon)	Yes; no
Flesch Reading Ease	Scored 0 to 100
Flesch–Kincaid Grade Level	Score corresponds with US education grade level
Functionality	
IMS Institute for Healthcare Informatics functionality score	Rated 1 (present) or 0 (absent) for the following functions: (1) inform, (2) instruct, (3) record, (3.1) collect data, (3.2) share data, (3.3) evaluate, (3.4) intervene, (4) display, (5) guide, (6) remind or alert, and (7) communicate
Quality	
Mobile App Rating Scale	19 items across four dimensions (engagement, functionality, aesthetics and information quality) rated on a 5-point Likert scale: 1 = inadequate, 2 = poor, 3 = acceptable, 4 = good, and 5 = excellent

Content-related information included the purpose of the app, a short summary, and whether it was related to genetics. We also determined the reading level for each app by copying the content into a Microsoft Word document and using two Flesch–Kincaid metrics (Flesch 1979; Kincaid et al. 1975). The Flesch–Kincaid Reading Ease score ranged from 0 to 100, with higher scores indicating that the material is easier to read (Flesch 1979). The Flesch–Kincaid Grade Level was also used, with scores referring to the equivalent grade level of education in the USA (Kincaid et al. 1975).

Two validated scales were used to assess functionality and quality, respectively. The IMS Institute for Healthcare Informatics functionality score (Parsippany 2013) helped to determine which functions were offered within apps. This scale consists of 7 items and 4 subcategories, which correspond with specific functions. Items are rated 1 if the function is present and 0 if it is not. The total score was generated by summing the items and ranged from 0 to 11. Scores between raters were cross-compared, and any disagreement was resolved through discussion.

As in other systematic app reviews (Ali et al. 2020; Gasteiger et al. 2021; Grainger et al. 2017; Kim et al. 2018; Talwar et al. 2019), the Mobile App Rating Scale (Stoyanov et al. 2015) was used to determine the quality of each app. The MARS consists of 19 items across four dimensions (engagement, functionality, aesthetics, and information quality), with each item rated on a 5-point Likert scale: (1) inadequate, (2) poor, (3) acceptable, (4) good, and (5) excellent. As in previous app reviews, the optional subscale for subjective quality was omitted to ensure that quality assessments were only objective (Ali et al. 2020; Bardus et al. 2016; Gasteiger et al. 2021; Kim et al. 2018).

Raters first watched the MARS training video on YouTube (Stoyanov 2016) and then independently rated each app. They answered the question: ‘Has the app been trialled/tested?’ by searching for published literature on evaluation (e.g. usability, satisfaction, effectiveness). Scores for each dimension were summed, and an overall mean score for quality was calculated. A mean score between the reviewers was then calculated.

### Descriptive analysis

Descriptive summary statistics were generated on applicable items. We conducted inter-rater reliability statistics for the MARS and the IMS Institute for Healthcare Informatics functionality scores, using IBM SPSS Statistics (version 23; IBM, Armonk, NY). We calculated intra-class correlation coefficients (ICCs) on all MARS items using absolute agreement 2-way mixed-effects, average-measures models (Shrout and Fleiss 1979). Cohen’s kappa was calculated for the IMS score, given that items are rated categorically (0 = no, 1 = yes). In order to determine the best-rated apps, we identified and cross-compared the highest scores across the MARS and IMS measures.

## Results

**Fig. 1:**
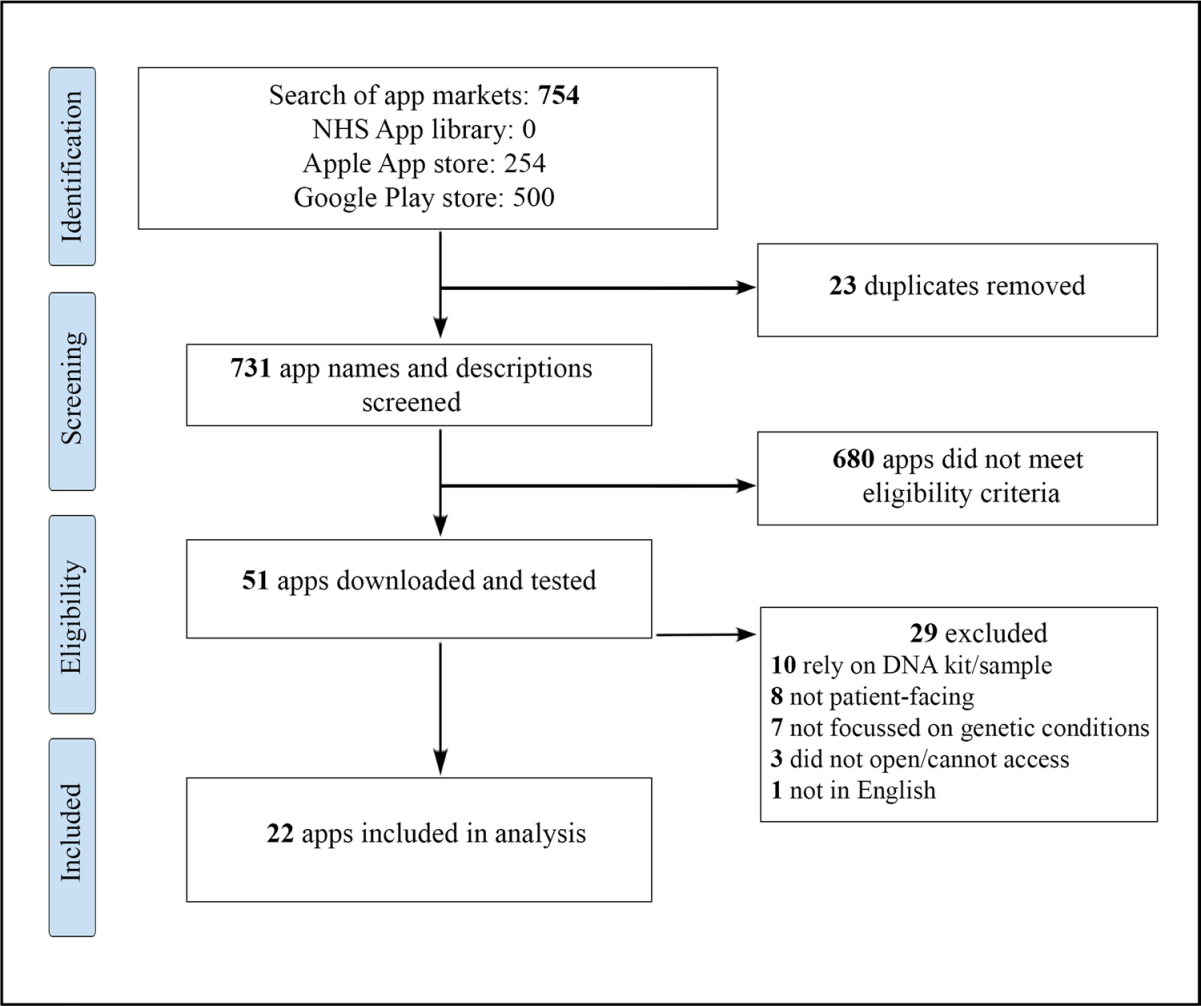
PRISMA flowchart depicting the app search and screening process

The search yielded a total of 754 apps across the Apple App and Google Play stores. None were identified from the NHS Apps Library. Twenty-three duplicates were removed, while 731 names and descriptions were screened, highlighting that 680 apps did not meet the eligibility criteria. A total of 51 apps were downloaded for a second screening; after which, 29 were removed for reasons such as relying on DNA kits/samples (*n* = 10), not being patient-facing (*n* = 8), not focussing on genetic conditions (*n* = 7), not opening or being unable to access (*n* = 3), and not being in English (*n* = 1). Twenty-two apps were consequently included in the review. Figure 1 presents the PRISMA flowchart summarising the search and screening process.

### Descriptive characteristics

Most (*n* = 20, 91%) of the 22 apps were available from the Google Play store, and 7 (32%) were available from the Apple store. Five (23%) were available from both stores. Of the 22 apps, the majority (*n* = 20, 91%) were free to download, seven (32%) of which had in-app purchases. There were two apps (9%) that required payment, costing £2.19 and £2.99. Of the 22 apps, only three (14%) were affiliated with a professional medical/health body or charity. The average user rating score was 2.8 of 5 (0 to 4.8), and six (27%) of the apps had not received any user ratings. The total number of user ratings was 303 (ranging from 1 to 123).

Of the 22 apps, 55% (*n* = 12) worked offline, and 5% (*n* = 1) asked users to enable notifications. None asked users whether they could work in the background. A privacy strategy was included in 36% (*n* = 8) of the 22 apps, with 23% (*n* = 5) asking for a password or login. Table 2 summarises some key characteristics.

**Table 2:** Key characteristics of the 22 reviewed apps

Characteristics	Number (%)
Purchase costs	
Free to download	20 (91)
In-app purchases	7 (32)
Costs to download	2 (9)
Clearly about genetics	
Yes	15 (68)
No	7 (32)
Affiliated with health body or charity	
Yes	3 (14)
No	19 (86)
Had a privacy strategy (e.g. login, policy)	
Yes	8 (36)
No	14 (64)
Enabled third-party sharing	
Yes	1 (5)
No	21 (95)

### Genomic content

All 22 apps aimed to educate/inform users, while 32% (7 of 22) analysed DNA samples, 23% (5 of 22) tracked or recorded data, and 18% (4 of 22) helped to assist diagnoses. None acted as reminder apps. When considering each app’s icon and name, it was clear that 68% (15 of 22) were about genetics or genomic conditions (see Table 2), with icons such as the helix or chromosome. On average, the apps had a Flesch Reading Ease score of 35 of 100 (range 8.9 to 76.8, SD: 16.8). The US reading age-grade level was on average 11.7 (grade 12), with content ranging from US school grades 7.9 to 17 (SD: 2).

### Functionality

The IMS Institute for Healthcare Informatics Functionality score was used to identify functions available within the apps. For the IMS scale; there was substantial agreement between the two raters’ scores of the Apple App store apps, *κ* = 0.694 (95% CI, 0.541 to 0.847), *p* < 0.000. There was almost a perfect agreement between the two raters of the Google Play apps, *κ* = 0.867 (95% CI, 0.791 to 0.943), *p* < 0.000.

**Fig. 2:**
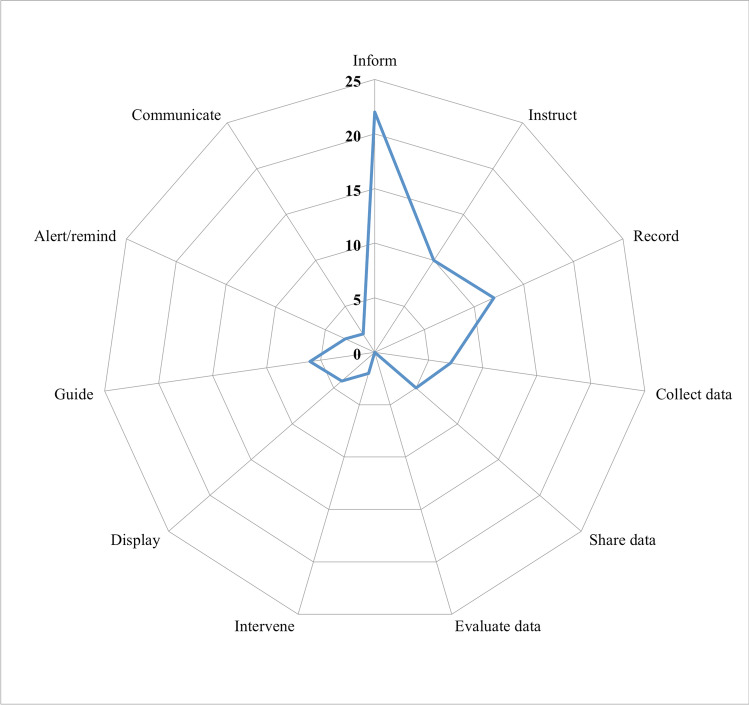
Radar graph showing the functions available in the reviewed genetic apps

The apps had an average of 3.3 functions each (SD: 2.6), ranging from 1 to 9 functions. As highlighted in the radar graph in Fig. 2, the three most common functions were inform (100%, *n* = 22), record (55%, *n* = 12), and instruct (45%, *n* = 10), while the least common were evaluate (0%), intervene (9%, *n* = 2), and communicate (9%, *n* = 2).

Only one app (Muhdo) had nine functions, and two (My Toolbox Genomics and Unlock MyDNA) had eight functions. Nine apps had only one function, which was providing information to users (see Supplementary Table 1).

### Quality

All apps were independently reviewed for quality by two raters using MARS. There was good agreement between the two raters for the Apple App store apps (ICC, 0.83; 95% CI, 0.744 to 0.878), *p* < 0.000. There was also a good agreement between the raters for the Google Play app (ICC, 0.85; 95% CI, 0.806 to 0.878), *p* < 0.000.

The mean overall total quality score was 3.2 ± 0.7 (of 5). Half (*n* = 11) of the apps met the minimum acceptability score of 3 (Stoyanov et al. 2015). The highest scoring apps were Bodyology DNA and My Toolbox Genomics, scoring 4.1. The lowest scoring app was 2.0 for genetic health disorders. Mean scores for engagement, functionality, aesthetic, and information quality were 2.5 ± 1.0, 4.3 ± 0.5, 3.1 ± 0.8, and 2.9 ± 0.9, respectively. Most apps scored the highest for functionality, with scores ranging from 3.0 to 4.8. The engagement was scored the lowest, with scores ranging from 1.2 to 4.0. None of the apps had been formally trialled or tested.

### Cross-comparing the apps

**Fig. 3:**
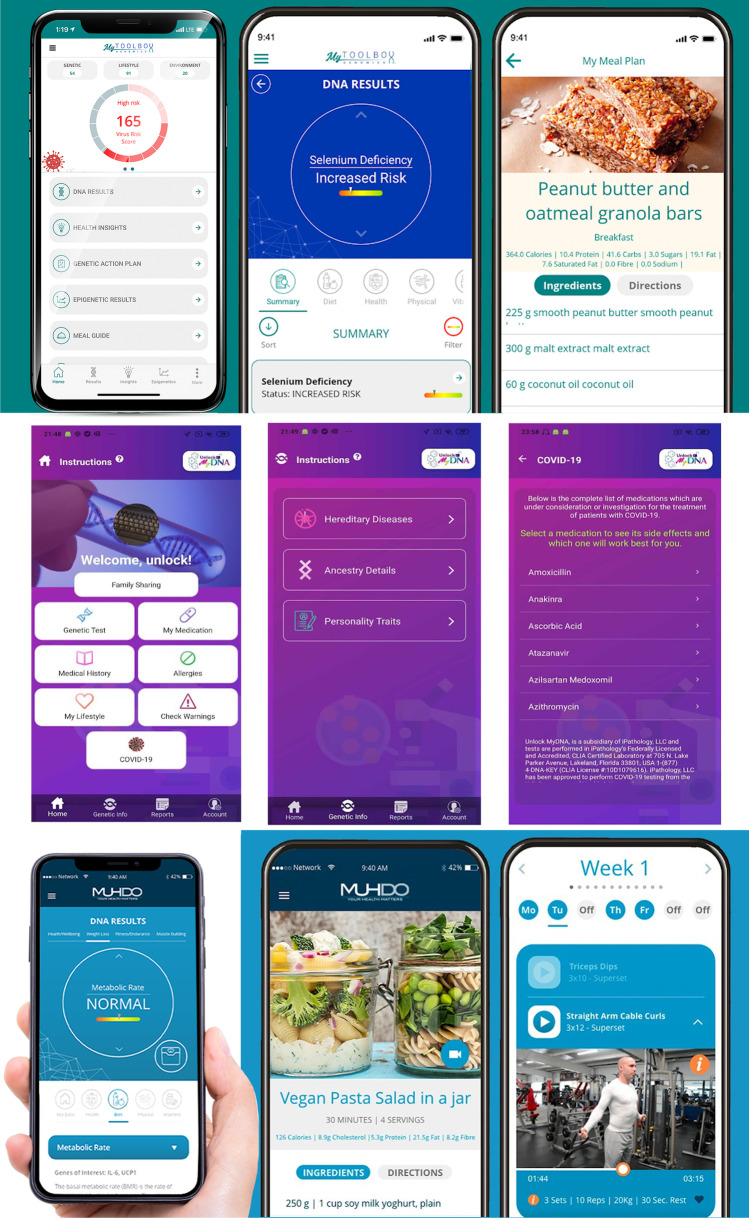
Top: the ‘My Toolbox Genomics’ app, showing personalised recommendations, workout and nutrition plans. Middle: images of the ‘Unlock MyDNA’ app showing potential risk and efficacy of medications. Bottom: the ‘Muhdo’ app, showing lifestyle tracking, meal guide, and training session features

The IMS and MARS scores were compared to identify the highest-performing genetic apps. Three apps were identified and are presented in Table 3 and Fig. 3. They include My Toolbox Genomics, Muhdo, and Unlock MyDNA. The My ToolBox Genomics and Muhdo apps both determined genetic health through results from a DNA sample and provided personalised recommendations, workout, and nutrition plans. Users could also book a doctor through the Muhdo app. The Unlock MyDNA app also analysed genetic data but functioned as a real-time personalised and predictive medical management, tracking, reporting, and notification system that alerts users to critical healthcare events (e.g. reactions to medicines).

**Table 3:** Highest scoring patient-facing genetic apps, when considering functionality and quality

App name	Market, cost	MARS*	IMS**	Description
My Toolbox Genomics	Apple, Google Play Free to download, in-app purchases	4.1	8	Users create a personal profile. Their genetic health is then determined through results from a saliva sample. The sample is analysed for 1000 genetic areas across 5 core areas: physical, diet, vitamins, health, and psychology. The app highlights genetic deficiencies and health risks. It also provides personalised recommendations, workout, and nutrition plans
Muhdo	Google Play Free to download	4.0	9	This app is very similar to My Toolbox Genomics. Users create a personal profile and upload their DNA kit files. The app analyses it for 1000 genetic areas and presents a report on 5 core areas: diet, physical, vitamins, health, and psychology. The app offers a personalised genetic action plan, lifestyle tracking, meal guide, and training sessions. Users can also book an on-call doctor
Unlock MyDNA	Google Play Free to download, in-app purchases	4.0	8	Users create a personal profile. By using their genetic history, the app functions as a real-time personalised and predictive medical management, tracking, reporting, and notification system that alerts users to critical healthcare events (e.g. adverse events related to over 145,000 medicines/supplements). It explores over 905,000 gene variants, identifies a patient’s personality traits, inherent behaviours, physical appearance, ancestry, a potential risk for diseases, hereditary precursors, and reaction to medications. Users can also order a DNA test kit through the app

^*^Overall mean score for the Mobile App Rating Scale (maximum score 5)

^*^IMS: overall score for the IMS Institute for Healthcare Informatics functionality score (range 0–11)

## Discussion

This app review examined patient-facing genomic and genetic apps available in the UK. Of the 22 apps reviewed, all intended to inform or educate users, while 32% also analysed genetic data, and 18% helped to diagnose genetic conditions. Most (68%) were clearly about genetic conditions, but only 14% were affiliated with a medical/health body or charity. Additionally, only 36% had a privacy strategy. Reading scores were low on average (35 of 100), with the average reading age being equivalent to US grade 12 (year 13 or sixth form in the UK). On average, the apps had 3.3 functions and a quality score of 3.2. Half met the minimum acceptability score for quality, but none had been formally evaluated.

From our search and screening process, it was clear that very few genetic/genomic apps available in the UK were patient facing. This was evident by only 22 of 731 genetic apps being included in our review. Similar findings were reported by Talwar et al. (2019), whereby 86.4% of their 88 reviewed apps targeted health professional students, and 78.5% did not focus on any specific diseases. Additionally, we found that the apps available in the UK were relatively simple in functionality, and half were low quality, meaning that they did not meet the MARS acceptability score. This highlights the opportunity for a new high-quality patient-facing genetic app to be developed in the UK.

Mobile health apps in general can have unclear and complex security measures. When digital platforms are aimed at nuanced population groups, factors associated with digital security should be of the utmost importance to creators. Dehling et al. (2015) carried out an overview of mobile health apps and identified that out of 17,979 reviewed, 95.63% (*n* = 17,193) posed potential information security and privacy infringement risks. A review of 20,000 medical and health apps highlighted that 45% rely on unencrypted communication, while 23% of personal data (e.g. passwords or location) is sent on unsecured traffic (Tangari et al. 2021). Another analysis that explored data security processes for mobile health apps raised serious concerns regarding privacy, with no privacy policy identified across 5903 of 20,991 (28.1%) apps reviewed (Tangari et al. 2021). This is not dissimilar to our findings, where only 36% (*n* = 8) of the apps reviewed included a privacy strategy, with 23% (*n* = 5) asking for a password or log in.

Poor privacy strategies may also deter engagement, as patients may wish to keep their use of genetic/genomic apps private. Genomics can reveal sensitive information regarding group ancestry, which can create blame aimed towards individuals and within population groups (de Vries et al. 2020). Discrimination is purported to affect a broad range of genetic conditions, encompassing incidences in the workplace, issues when seeking insurance, and general social situations (Williams et al. 2010). As genetic testing is becoming more prevalent and accurate due to an ever-growing diagnostic capability, it is apparent that there are emerging ethical challenges associated with advancing technology (Chapman et al. 2020). Some countries have responded by enacting specific policies, such as the USA’s Genetic Information Non-discrimination Act (GINA) ('The Genetic Information Nondiscrimination Act: A First Step Toward Protecting Americans From Misuse of Genetic Information' 2009). Its goal was to ensure the law protected those who wanted to take advantage of genetic testing in clinical and research settings without experiencing genetic discrimination. Within communities where consanguineous marriage is practised, there are many factors that can affect the engagement with clinical genetics, with perceived religious and cultural barriers being at the core (Alkuraya 2013). Genetic risks have been inappropriately reported in the past, fuelling negative connotations and increasing stigma and sensationalist views regarding consanguinity (Bhopal et al. 2014). As such, it is imperative that any apps aimed at improving genetic literacy have adequate security and privacy settings.

It is apparent that, at times, there can be conflicting information regarding the risk of genetic disorders associated with consanguinity depending on the source, which can cause confusion amongst communities and a lack of trust in healthcare professionals (Darr et al. 2016). Mobile apps that are affiliated with a registered charity or body are more likely to contain accurate, evidence-based information, which, in turn, has the potential to increase engagement and thus, its impact. Of our 22 apps reviewed, only 14% (*n* = 3) were affiliated with a registered charity or health organisation, suggesting that in the broader context of the design of the authors’ app such an association would be beneficial.

Similarly, none of the 22 apps we reviewed had been verified by evidence in published scientific literature. This brings the reliability and quality of the information into question. In the context of a patient-facing app, it can be argued that the need for high quality, accurate, and culturally sensitive information is an absolute necessity in order to promote engagement and engender trust. Involving future end users in the codesign process is more likely to ensure that the content is appropriate, acceptable, and the final app is utilised. During the design of a health app for Chronic Obstructive Pulmonary Disease (COPD), researchers found that target users, researchers, and developers should be involved at every stage of app development, using an agile approach, including the building of a prototype app, which should then be tested in controlled settings as well as in the wild (Davies et al. 2020).

The language presented in the genetic apps may also limit accessibility and acceptability, particularly for users for whom English is not their native language. In our review, all of the apps intended to inform/educate users; however, the mean reading score was only 35 of 100, with language written at the level of US grade 12 (17-year-olds). This finding was concerning and highlighted an obvious mismatch between the general public’s literacy skills and the content presented and maybe of particular concern for a user group where English is not their native language. In 2011, the UK government conducted a survey on adult literacy and found that one in seven adults had literacy levels equivalent to that of a 9 to an 11-year-old child (Department for Business Innovation & Skills 2012). An OECD survey in 2016 found that 9 million working adults in England have low literacy (or numeracy) skills, including those with university qualifications (Organisation for Economic Co-operation and Development 2016). More recently, NHS Digital conducted an informal audit on NHS website content, finding that the average reading age is around 16 years, with 80% of adults not able to read at this level (NHS 2021; Robinson and Savic 2019). Ultimately, this mismatch can have important and negative consequences on patients, including decreasing health literacy or creating misunderstanding and unnecessary confusion about health information. Adults with low literacy may also be deterred from using genetic apps, thus contributing to inequitable access between users and nonusers.

## Implications

Currently, there are few high-quality patient-facing genetic/genomic apps available in the UK. This warrants an opportunity for the design, development, and evaluation of a high-quality smartphone app, developed in partnership with groups most at-risk of genetic disorders.

It is imperative that the design of future genetic apps prioritises privacy and security, especially when storing and recording health, genetic, and personally identifiable data. Collaboration with future end users may help to provide insight as to whether affiliating the app with genetics (e.g. through the name and icon) may facilitate or deter use. This is because some cultural groups and patients may experience stigma or discrimination associated with genetic disorders (Williams et al. 2010). Affiliating the app with a reputable organisation (e.g. NHS) might also help to normalize genetic conditions and help to increase trust in the content being presented. Collaboration and partnership with cultural groups can also help to ensure cultural accessibility, including offering content in a user’s local language and presenting sensitive content in a culturally appropriate manner. This could be important for helping overcome the previously reported religious and cultural barriers to clinical genetics (Alkuraya 2013).

Future genetic apps also need to incorporate language that is easy to understand. The importance of accessible health information for health literacy has already been recognised by various organisations in the UK, including the NHS and Health Education England. The NHS Digital Service manual (NHS 2021) and Health Literacy ‘How to’ Guides (NHS Health Education England, n.d.) should therefore be used to develop appropriate content. Reading tools, such as the Flesch–Kincaid metrics (Flesch 1979; Kincaid et al. 1975), can be used in conjunction with the language guides to assess and predict readability.

## Strengths and limitations

Limitations of the review must be acknowledged. First, apps only available in the UK at one time point were included. This may mean that the apps are no longer available in the future or have been updated. Additionally, only English apps were reviewed. Lastly, apps were identified from the NHS Apps Library and the Apple App and Google Play stores as the latter are the dominant operating systems. This means that apps only mentioned in academic literature, released privately, or available on Amazon or Windows were not included.

Strengths include the addition of measures, such as the Flesch–Kincaid metrics and the IMS, which have not been employed in previous app reviews (Ali et al. 2020; Bondaronek et al. 2018; Talwar et al. 2019). These measures help to explore other important aspects of health apps, such as the readability of content and functionality. A validated scale, MARS, was also used to explore quality. Lastly, the search was extensive, by including paid apps and those which also include DNA samples/kits.

## Conclusion

Many genetic/genomic apps were available across two UK app stores. However, the majority were not patient facing, and many relied on additional DNA testing kits. Of the 22 apps that were reviewed, half did not meet the minimum acceptability criteria, and on average, they had few functions. Only three apps were affiliated with a registered charity or healthcare organisation, and none had been formally trialled or tested. Increasing awareness and education related to genetic conditions and the potential risks associated with consanguineous relationships are important but must be done with the input from future end users, ensuring that content is accessible, sensitive, and culturally appropriate. This review ultimately highlights an opportunity for an accessible, culturally sensitive, evidence-based app to be developed and evaluated, which could improve genetic literacy within patient populations and specific communities.

## Supplementary Information

Below is the link to the electronic supplementary material.

Supplementary file1 (DOCX 92 kb)

## Data Availability

Not applicable.
