# Supplementary material for: Patient-facing genetic and genomic mobile apps in the UK: a systematic review of content, functionality, and quality
Source: J Community Genet. 2022 Feb 19;13(2):171–82. doi: 10.1007/s12687-022-00579-y (PMC8941009; doi:10.1007/s12687-022-00579-y)
Supplement: Supplementary file 1 — Supplementary file1 (DOCX 92 kb) [file 12687_2022_579_MOESM1_ESM.docx]

**Supplementary file**

Table S1. Summary of the reviewed apps, reading scores and IMS.

| Name | Version | Developer | Market/s | Cost* | Reading score; reading age | Purpose | IMS** |
| --- | --- | --- | --- | --- | --- | --- | --- |
| Genetic disorders and syndromes pocket | 2.1 | Tran Tuan | Apple | 2.99 | 20.4; 12.5 | Inform/ educate | 2 |
| Parent project MD | 6.62.0 | Mobile roadie | Apple | Free | 28.9; 15.6 | Inform/ educate | 3 |
| Genomapp. Squeeze your DNA | 6.47 | RF Developments | Apple; Google Play | Free | 18.4; 12 | Inform/ educate, analyse data | 6 |
| Smart DNA MyGenomeBox | 2.1 | MyGenomeBox | Apple; Google Play | Free | 8.9; 17 | Inform/ educate, analyse data, track/ record data, assist diagnosis | 5 |
| Genes & Diseases | 3.4 | Omelette, Inc. | Apple; Google Play | Free | 46.1; 11 | Inform/ educate | 3 |
| Bodyology DNA | 1.0.0 | Muhdo | Apple; Google Play | Free | 37; 12.4 | Inform/ educate, analyse data, track/ record, assist diagnosis | 6 |
| My Toolbox Genomics | 1.0.6 | Toolbox Genomics, Inc | Apple; Google Play | Free | 47.6; 8.3 | Inform/ educate, analyse data, track/ record, assist diagnosis | 8 |
| Muhdo | 3.1.51 | Muhdo | Google Play | Free | 42.7; 12 | Inform/ educate, analyse data, track/ record | 9 |
| Genetic Disorders | 1 | Koby Apps | Google Play | Free | 17.9; 12 | Inform/ educate | 3 |
| Porphyria Disease | 1.0.0 | bedieman | Google Play | Free | 39.1; 12 | Inform/ educate | 1 |
| Unlock MyDNA | 1.1.0 | Unlock MyDNA | Google Play | Free | 39.2; 12 | Inform/ educate, analyse data, track/ record | 8 |
| Thalassemia Disease | 1.0.0 | bedieman | Google Play | Free | 19.1; 12 | Inform/ educate | 1 |
| Recognize Hemophilia Disease | 3.0.1 | Media Clinic | Google Play | Free | 29.3; 12 | Inform/ educate | 1 |
| Recognize Albinism Disease | 3.0.1 | Media Clinic | Google Play | Free | 24; 12 | Inform/ educate | 1 |
| Rare Disease Identification | 1.0 | Pranav Sriraman and Ananth Subramanya | Google Play | Free | 22.3; 12 | Inform/ educate, assist diagnosis | 1 |
| Hemophilia Disease | 1.0.0 | bedieman | Google Play | Free | 30.8; 12 | Inform/ educate | 1 |
| Geneus DNA | 2.4.0 | Geneus Genetics Co.,Ltd | Google Play | Free | 59.1; 10.9 | Inform/ educate, analyse data | 5 |
| Genetics- Note | 1.1 | DhadbadatiApps | Google Play | Free | 62.1; 7.9 | Inform/ educate | 1 |
| Genetics Dictionary | 0.0.7 | techhuw | Google Play | Free | 41; 11.3 | Inform/ educate | 1 |
| Genetics Dictionary | 2.1.1 | Apps Artist | Google Play | Free | 41.9; 11 | Inform/ educate | 1 |
| Genetic Heredity Calculator | 1.1.1 | Thrice Solutions | Google Play | Free | 76.8; 8.2 | Inform/ educate | 3 |
| Genetic Health Disorders | 1.0 | Koby Apps | Google Play | 2.19 | 17.9; 12 | Inform/ educate | 3 |

*Currency is in GBP
**IMS: Overall score for the IMS Institute for Healthcare Informatics functionality score (range 0-11).

Table S2. Mobile App Rating Scale dimensions and total scores for the reviewed apps.

| Name | Engagement | Functionality | Aesthetic | Information Quality | Total |
| --- | --- | --- | --- | --- | --- |
| Genetic disorders and syndromes pocket | 2.2 | 4.8 | 2.8 | 3.8 | 3.4 |
| Parent project MD | 2 | 3.8 | 3.3 | 3.9 | 3.2 |
| Genomapp. Squeeze your DNA | 3.2 | 4.5 | 4.0 | 3.8 | 3.9 |
| Smart DNA MyGenomeBox | 4.2 | 4.0 | 4.0 | 3.5 | 3.9 |
| Genes & Diseases | 2.2 | 4.0 | 4.0 | 4.0 | 3.6 |
| Bodyology DNA | 3.5 | 4.4 | 4.3 | 4.3 | 4.1 |
| My Toolbox Genomics | 3.6 | 4.4 | 4.2 | 4.2 | 4.1 |
| Muhdo | 4 | 4.1 | 4.0 | 3.7 | 4.0 |
| Genetic Disorders | 1.9 | 3.0 | 2.2 | 1.6 | 2.2 |
| Porphyria Disease | 1.6 | 4.5 | 2.5 | 2.1 | 2.7 |
| Unlock MyDNA | 4 | 4.6 | 3.8 | 3.4 | 4.0 |
| Thalassemia Disease | 1.7 | 4.4 | 2.5 | 2.1 | 2.7 |
| Recognize Hemophilia Disease | 1.9 | 4.6 | 2.3 | 2.5 | 2.8 |
| Recognize Albinism Disease | 1.7 | 4.5 | 2.3 | 2.5 | 2.8 |
| Rare Disease Identification | 1.4 | 3.9 | 2.3 | 2.1 | 2.4 |
| Hemophilia Disease | 1.7 | 4.6 | 2.5 | 2.1 | 2.7 |
| Geneus DNA | 3.5 | 4.8 | 4.2 | 3.2 | 3.9 |
| Genetics- Note | 2.1 | 4.5 | 3.0 | 1.8 | 2.9 |
| Genetics Dictionary | 1.6 | 4.8 | 2.7 | 2.1 | 2.8 |
| Genetics Dictionary | 1.8 | 4.8 | 2.3 | 2.1 | 2.8 |
| Genetic Heredity Calculator | 3 | 4.8 | 2.3 | 2.6 | 3.2 |
| Genetic Health Disorders | 1.2 | 3.1 | 1.8 | 1.7 | 2.0 |
